# Targeting Gene Transcription Prevents Antibiotic Resistance

**DOI:** 10.3390/antibiotics14040345

**Published:** 2025-03-27

**Authors:** Paul F. Agris

**Affiliations:** Department of Medicine, Duke University School of Medicine, Durham, NC 27710, USA; paul.agris@duke.edu; Tel.: +1-919-961-4274

**Keywords:** antibiotic resistance, discovery strategy, novel mRNA target, multi-targeting

## Abstract

Innovative strategies are needed to curb the global health challenge of antibiotic resistance. The World Health Organization predicts that antibiotic resistance could lead to millions of deaths annually. Pharmaceutical experience has shown that modest alterations of commonly-used broad-spectrum antibiotics readily elicit resistant strains. Thus, continued simple iterative improvements on current antibiotics are not sustainable. Traditional strategies target single sites with the goal of a broad-spectrum antibiotic. In comparison, a novel strategy targets multiple sites in single- or multidrug-resistant Gram-positive bacterial pathogens. The objective is to exploit the mechanisms by which pathogenic bacteria require genes for transcriptional regulation. Transcription regulatory factors can be manipulated and their functions disrupted to hamper bacterial viability. Some transcription factors regulate one or more steps in metabolic pathways. Transcription factors are not always proteins; some are small-molecule metabolites triggering genetic functions through riboswitches, and others are RNAs. Novel agents have been discovered with computer-simulated docking to an unusual transcription regulatory site in nascent bacterial mRNA. These compounds exhibit innovative chemistries and modes of action that inhibit bacterial growth by binding to and blocking critical Gram-positive mRNA functions. The tRNA-dependent transcription regulation of amino acid metabolism illustrates the possibilities of novel strategies to inhibit antibiotic-resistant growth and thwart the emergence of novel resistant strains.

## 1. Introduction

### 1.1. Present Challenges from Antimicrobial Resistance and Traditional Pathways to Antibiotics

By 2050, antimicrobial resistance (AMR) could cause 10 million deaths worldwide each year [1]. Most researchers pursuing new antibiotics have followed traditional discovery and development pathways with no new insights. The implications of not pursuing an innovative strategy for antibiotic discovery are a sustained and dangerous epidemic of antibiotic resistance. AMR has been escalating for decades in the U.S. [2]. The facts are overwhelming and disheartening. Each year, more than 2.8 million people become infected with antibiotic resistant bacteria in the U.S., and at least 35,000 people die as a direct result of these infections [3]. Antibiotic resistance is a leading cause of death of children younger than 5 years. The infection rate of the antibiotic-resistant Gram-positive bacterium *Clostridia difficile*, or *C. diff*., was 500,000 just among patients in the U.S. [4], and it is designated an “urgent” threat. Within 30 days of their diagnosis, some 29,000 patients died in the U.S. in 2020. Other Gram-positive bacterial infections are “serious” threats, such as *vancomycin-resistant Enterococci (VRE)*, *methicillin-resistant Staphylococcus aureus (MRSA)*, and antibiotic-resistant *Streptococcus pneumoniae*. Add *C. diff.* to these numbers and the totals are 3 million infections and 48,000 deaths in the U.S. each year for many years. Gram-positive bacteria are the most common bacteria to colonize implantable medical devices. Over seven million Americans currently have joint implants, mostly one million knee and hip implants each year. Some 370,000 cardiac pacemakers are implanted in U.S. patients annually. The infection takes the form of a biofilm.

Biofilms are inherently challenging to treat because the bacteria protect themselves with a gel, an extracellular polymeric substance (EPS; polysaccharides, lipids, nucleic acids, proteins, lipopolysaccharides, and minerals) that is difficult for traditional antibiotics to penetrate. Antibiotic-resistant biofilms are even more challenging to manage. Biofilms are one of the three basic mechanisms bacteria utilize for resistance. The two additional mechanisms are removal of the antibiotic with existing efflux pumps before the drug can be effective and enzymatically altering the target site(s) with new chemistries [5]. Long-established research strategies that alter existing antibiotics for the development of new drugs have demonstrated that soon after the introduction of the modified antibiotic, resistant strains emerge. Antibiotic-resistant clinical strains are found for every chemical class of antibiotic, no matter the mechanism of action of the antibiotic [6]. Thus, new strategies are urgently needed to combat antibiotic resistance in bacterial planktonic and biofilm growth.

### 1.2. Proteins Versus RNA Targets

Proteins have 20 plus distinctive amino acid chemistries and a diversity of structures that could credibly be targeted. Protein transcription factors (PTFs) regulate RNA transcription by recognizing and binding to the promotor regions of DNA or to nascent mRNAs. PTFs are activated by specific small-molecule metabolites or environmental products. PTFs either inhibit or activate targeted genes. The majority of PTFs are inhibitors of transcription, altering the DNA conformation and obstructing the progression of RNA polymerase. Many PTFs affect multiple genes, and some PTFs target a single gene. PTFs bind DNA in a specific sequence context, and a few PTFs recognize DNA-modified bases [7]. Cancers are characterized by having abnormal or unregulated transcription factors; unregulated PTFs associated with cancers have become targets of intervention [8]. Thus, a common therapeutic strategy is to target the oncogenic dysregulation of genes with small molecules, yet PTFs are intrinsically disordered proteins lacking well-defined sites to which small molecules can bind [9,10].

Ribonucleic acids, RNAs, are an important and novel target in combating AMR. Unfortunately, there is a symmetry about RNA chemistries that does not generate confidence in researchers designing small-molecule antibiotics that target nucleic acids, even with the use of artificial intelligence. Like proteins, RNA is a single-stranded heteropolymer but is composed of only four quite similar purine and pyrimidine chemistries on a backbone of ribonucleoside phosphates. RNAs transcribed from DNA are identical to one of the DNA double strands and complementary to the other—except for uridine replacing deoxythymidine and ribose replacing deoxyribose. There are many types of RNA, some with only a few nucleosides and some with thousands. RNAs transcribed from DNA have many structural and functional roles in all organisms. Due to the success of the COVID-19 vaccines, messenger RNAs, mRNAs, continue to be investigated as vaccines for viral infections. mRNAs have post-transcriptional sites for modifications, processing information for the sizing of the mRNA, and protein-binding sites for the regulation of transcription or translation. Within the non-coding regions of the nascent mRNA sequences are found the chemistry and structural information for the ligand binding that regulates transcription.

## 2. Targeting RNA Function

### 2.1. RNA Transcription Factors

Transcription factors are not always proteins, some are RNAs. RNA transcription regulatory elements in bacteria consist of three classes. One class of RNA regulatory elements is the RNA riboswitch [11]. Riboswitches are typically located in untranslated, non-coding regions of mRNAs. They are activated by small-molecule metabolites specific to the gene or genetic pathway. Upon binding the metabolite, the RNA riboswitch conformation is altered, resulting in transcription or translation initiation or termination. Metabolites that effect translational riboswitches act in *cis* and modulate the translation initiation. Each riboswitch regulates a particular mRNA. Small-molecule antibiotic discovery and development have been initiated for a number of riboswitches [12,13] A second class of RNA transcription factors is the long non-coding RNAs, lncRNAs [14]. lncRNAs have necessary regulatory functions. They regulate transcription in *cis* and in addition act as transcription-stabilizing elements for dosage-sensitive genes that encode transcription factors [15]. Bacterial RNA transcription factors can be used as targets for the treatment of antibiotic resistance. New antibiotics that target RNA transcription factors intrinsic in bacterial mRNAs could not only inhibit bacterial growth but also impede the evolution of resistance.

In this brief Review, four criteria are proposed to discover and develop new strategies for novel antibiotics that inhibit resistance and the emergence of resistance. First, there is a need to develop understanding of entirely new targets critical to bacterial viability with unusual chemistries and shapes, targets not found in the human host [6]. Second, research should focus on a narrow landscape of infecting bacteria. A new antibiotic will sacrifice breadth for efficacy. Third, new antibiotics need to be formulated with novel chemistries and shapes that complement only the new targets and penetrate biofilm EPS barriers. Their mechanism of action needs to be characterized. Established antibiotics lack targeting and disperse to unintended sites with unintended consequences. The result is that sub-lethal concentrations attack the target site [16]. Fourth, a single antibiotic needs to target multiple bacterial functions, and only bacterial functions. By multi-targeting, the new antibiotic has the advantage of concurrently shutting down a few bacterial functions for which simultaneously occurring bacterial mutations toward resistance would be statistically improbable. These antibiotics would target a family of mRNAs from different genes.

### 2.2. A Novel Perspective

**Figure 1:**
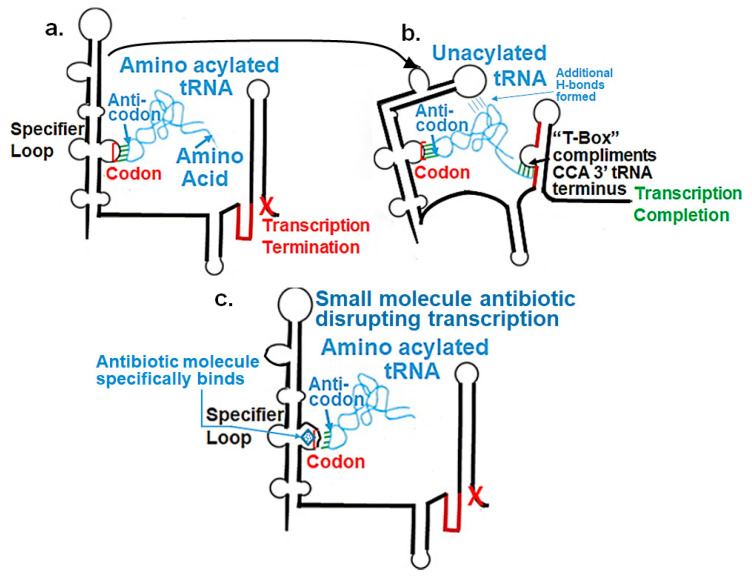
tRNA-dependent gene regulation of transcription. The 5′UTR of nascent mRNAs for the aminoacyl-tRNA synthetase genes of Gram-positive bacteria function as regulators of gene transcription. (**a**) Aminoacylated tRNA is bound by the 5′UTR specifier loop through its anticodon binding to the cognate codon. The nominal three-base interaction of anticodon/codon binding is many times expanded to a four-base interaction with the invariant U_34_. However, the aminoacyl-tRNA does not bind stably to the 5′UTR because other interactions with the mRNA are not possible, particularly the aminoacylated 3′CCA terminus with the T-Box UGG sequence. Transcription is terminated. (**b**) Unacylated tRNA binds to the cognate codon in the specifier loop. The unacylated 3′CCA terminus is free to bind to the T-Box UGG. The nascent mRNA changes its conformation resulting in a stabile tRNA–mRNA complex. Transcription is completed. (**c**) Small-molecule intervention inhibits specifier loop–codon interaction with tRNA’s anticodon by deforming the specifier loop. Transcription becomes terminated because the anticodon of unacylated (or acylated) tRNAs cannot bind the specifier loop codon (drawn from reference [19]).

By employing the four criteria, an entirely new target critical to bacterial viability has been described as having unusual chemistries and shapes, not found in the human host [17,18]. tRNA-dependent gene regulation functions as a third class of RNA transcription regulatory elements. tRNA-dependent gene regulation controls Gram-positive bacterial gene expression by controlling whether nascent mRNA transcription will be completed. Specific tRNAs operate as ligands binding the 5′-untranslated regions (5′UTR) of nascent mRNAs. Almost all Gram-positive bacteria, and only Gram-positive bacteria, have this regulatory element; it is not found in Gram-negative bacteria [18]. Many of the bacterial genes for amino acid metabolism are controlled by tRNA-dependent regulatory elements. Most of the 20 aminoacyl-tRNA synthetase genes are under tRNA-dependent transcription control, and the cognate tRNA isoacceptor acts as the controlling ligand [17,18]. The nascent mRNAs have common 5′-structural elements called “specifier loops”, which have similar adenosine-rich sequences but with identifying specific codon sequences. In the first step of transcription control, these codon sequences bind the cognate anticodons of the unacylated tRNAs. Then, other structural areas of the nascent mRNA bind the remote domains of the tRNA, such as the unacylated 5′-terminal CCA sequence to the UGG “T-Box” in the nascent mRNA. The nascent mRNA conformation is altered, allowing transcription to proceed. In contrast, aminoacyl-tRNA binds to the specifier loop codon but cannot achieve the required mRNA conformational fold in binding to other nascent mRNA sites. Aminoacyl-tRNA, having a cognate anticodon, does not activate the transcription to completion (Figure 1).

Finding an efficacious antibiotic that targets tRNA-dependent gene regulation is a formidable task. In contrast to most major pharmaceutical research developing antibiotics against Gram-negative as well as Gram-positive bacteria, the second criterion foregoes breadth to achieve efficacy and the ability to thwart the emergence of resistance. Thus, we chose to focus our research on a controlled landscape of known antibiotic-resistant Gram-positive bacteria recognized as posing urgent threats [20,21,22], for instance, *Staphylococcus* spp., (MRSA), *Streptomyces* spp. (macrolide-resistant *Streptococcus pneumoniae)*, and *Clostridioides* spp. *(Clostridium* spp.; resistant to tetracyclines, erythromycin, clindamycin, penicillin, cephalosporins, etc.). The tRNA-dependent transcription regulation mechanism in Gram-positive pathogens exhibits a number of characteristics that prove advantageous to drug target development. For instance, its function in regulating “housekeeping” genes is a proven strategy that novel antibiotics will abrogate gene expression and be lethal. The parent compound targeting tRNA-dependent transcription regulation, PKZ18, is considerably active against seven Gram-positive strains [21]. PKZ18 is chemically and structurally distinct from known antibiotics. The PKZ18 chemical family consists of a benzene ring and a thiazole ring connected to a norbornane ring via a peptide link. The benzene and thiazole rings have attached hydrophobic groups, while the norbornane ring has a carboxylic acid group. The benzene, thiazole, norbornane, and attached groups have been explored as to whether they could be chemically altered to benefit antibiotic activity [20,21].

The PKZ18 antibiotic family of compounds lacks activity against Gram-negative bacteria (Table 1). In contrast to a riboswitch target that occurs only once in an organism, a PKZ18 family compound targeting the conserved structural elements of tRNA-dependent transcription regulation deforms multiple specifier loops concurrently, repressing a considerable number of essential genes.

To employ the third condition, new antibiotics were formulated with novel chemistries and shapes that complement the new targets to understand the mechanism of action and determine whether the novel antibiotic penetrates biofilm EPS barriers. Computer molecular dynamics is increasingly useful in drug development to simulate the best compound configuration and chemical space that complements the target. Thus, to embark on the third criteria, simulated molecular dynamics (MD) was used to search more than a million compounds having RNA-binding potential [15]. In searching these compounds using MD, novel chemistries and shapes were discovered that complement the new mRNA specifier loop target. More recently, starting with the parent compound, the PKZ18 family has been extended into new compounds with new antibiotic activities using three structural sites: the norbornane, the thioazol ring, and the benzene (Table 2).

**Figure 2:**
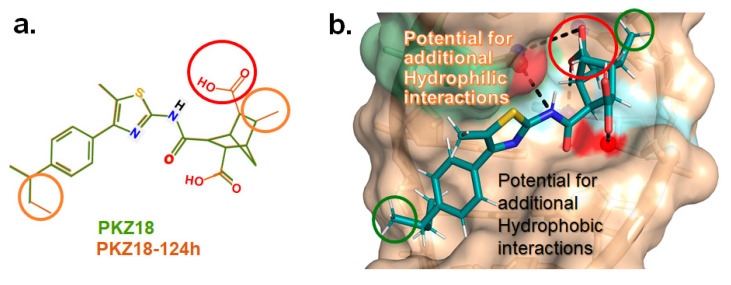
Specifier loop hydrophilic and hydrophobic surface areas to which PKZ18 binds. (**a**) The chemical structures of PKZ18 and the computer-simulated PKZ18-124h are superimposed. (**b**) The surface areas of the specifier loop tRNA-dependent regulatory element were interrogated with computational molecular modeling with the help of X-ray crystallographic and NMR information (Protein Data Bank, 4MGN, 4JRC, 4LCK [24,25]). A computational model of PKZ18-124h bound to the specifier loop yielded a ∆G = −7.1 binding energy. Computational molecular dynamics converted PKZ18 to PKZ18-124h for a stronger ligand binding to the surface of the Specifier Loop. The result was a hydrophilic addition of a carboxylic acid circled in red. Also resulting was hydrophobic additions that are circled in orange. For molecular docking, a target molecule considered inflexible requires minimal refinement at the site of the ligand binding [26]. Though having stable binding sites, mRNA is a dynamic molecule. Therefore, it is expected that a degree of flexibility be allowed in the mRNA specifier loop. To assess the binding strength of PKZ18 analogs, the dynamics of NMR structures were included in the calculations [2KHY and 2KZL [27,28]) (Eric Jestel and Sweta Vangaveti pre-publication communication).

The chemical alterations are designed to minimize the negative impact of the structural and chemical requirements of the parent compound’s activity [20] and complement the chemistry and structure of the target pocket of the mRNA specifier loop (Figure 2). An analog of the parent PKZ18, PKZ18-22 was most effective at penetrating the biofilm EPS [22]. PKZ18-22 inhibited the growth of *S. aureus* in biofilms and was 10-fold more potent than vancomycin. It was also synergistic with the antibiotics gentamicin and rifampin.

In applying the fourth criteria, a single antibiotic would be fashioned to target multiple bacterial functions. In contrast to commonly employed antibiotics, the advantage of a new antibiotic multi-targeting some 10–20 bacterial functions would be their simultaneous disruption. Bacterial antibiotic resistance at all of these sites would require concurring mutations and be statistically improbable. The analog of the PKZ18 parent compound, PKZ18-22, disrupted the aminoacyl-tRNA synthetase gene expression of 8 of 12 genes in MRSA [21]. Most of the aminoacyl-tRNA synthetase genes are tRNA-dependent controlled genes [18]. Other 5′-UTR regulated genes were not affected. An almost undetectable resistance was observed. MRSA exposed to MIC levels (64 µg/mL) of PKZ18-22 did not form resistant colonies, with only one colony recovered from a total of 1.8 × 10^11^ CFU plated, a resistance frequency of 5.6 × 10^12^. In comparison, gentamicin (20× MIC against MRSA) exhibited a resistance frequency of 1.6 × 10^7^, further supporting the existence of multiple targets for PKZ18 analogs [20,21]. PKZ18 is moderately cytotoxic at the MIC [20]. Against human cells in culture, the toxicity of the analog PKZ18-22 was observed at 2- to 4-fold higher concentrations than the MIC, thus being less toxic than the parent PKZ18 [20,21]. PKZ18 analogs alone or as a topical formulation could be a potent therapeutic for skin infections caused by Gram-positive bacteria such as MRSA. In dermal toxicity testing on mice by a contract research organization, PKZ18-22 exhibited a barely perceptible, slight erythema for 22–30% of the mice at the site of application. Redness resolved within 2–3 days for all mice. No dermal scores were indicative of irritation at the dose application site; all other observations and measurements at even the highest dose were normal. No acute toxicity was observed; no abnormalities were identified in a gross necropsy even at concentrations >100 times the MIC.

Analogs with both improved efficacy and a larger therapeutic window of cytotoxicity would be clinically beneficial. In order to yet again increase the binding of PKZ18 analogs to the specifier loop and decrease the MIC without increasing the toxicity of the compounds, the chemical structure of PKZ18-22 was altered using computer molecular design to complement the hydrophobic and hydrophilic surface areas of the specifier loop (Figure 2). One of the PKZ18 analogs, PKZ18-124h (Figure 2a), exhibited the highest molecular docking score to the specifier loop, predictive of its binding strength [23]. Relative to PKZ18, PKZ18-124h has an additional carboxylic acid moiety and methyl group on the norbornane ring, and the isopropyl group on the benzene is replaced by an isobutyl group (Figure 2). The binding of PKZ18-124h improved upon PKZ18 and analogs because it has an additional hydrophilic interaction and two additional hydrophobic interactions with the platform of the specifier loop (Figure 2b).

## 3. Discussion and Conclusions

The PKZ18 family of compounds has demonstrated significant antibiotic activities against Gram-positive bacteria [20,21,22]. Rather than a broad-spectrum antibiotic targeting Gram-negative and Gram-positive pathogens together, PKZ18 antibiotics multi-target a specific transcription regulatory element in multiple Gram-positive mRNAs. The mRNAs are transcribed from genes responsible for amino acid metabolism and are inhibited by PKZ18-22 [21]. One advantage to the tRNA-dependent transcription regulatory element being an antibiotic target is that it is found only in Gram-positive mRNAs, not in the human host. Computational docking and molecular dynamics recently has reduced investments and driven drug discovery. Our use of computational drug discovery has yielded PKZ18 analogs with superior docking scores to those of PKZ18 and PKZ18-22.

Pharmaceutical companies have tried to modestly alter old strategies whereby the commonly used broad-spectrum antibiotics against both Gram-positive and Gram-negative bacteria target and inhibit a single function critical to the viability of a wide range of disease-causing bacteria. The industry argues that antibiotics that act against specific infections have very small profit margins; broad-spectrum antibiotics are more profitable. With small profit margins, innovative drug research and development is prohibitively expensive. However, when a parent is confronted with the antibiotic resistance of their child’s infection, the cost is immaterial to saving that child’s life. Over the last two decades, when a prior strategy is employed in altering a common broad-spectrum antibiotic, an ever more rapid emergence of bacterial resistance is observed in the human population. The history of antibiotic-resistant bacterial strains appearing in the human population is commensurate with the amplified use of an antibiotic, for instance, in animal feed. Antibiotic resistance has been recognized as a continually reemerging infectious disease for decades, but it has been difficult to attain traction with new antibiotics.

New perspectives, novel targets and chemical entities, are important to discovering antibiotics that inhibit Gram-positive infections and the emergence of resistant strains. A unique strategy was employed and was successful in inhibiting the growth of Gram-positive pathogens in liquid media and biofilms. Using small molecules to target tRNA-dependent transcription regulation, a family of compounds was found to disrupt bacterial growth by multi-targeting the tRNA-controlled gene expression of aminoacyl-tRNA synthetases, for instance, *S. aureus*, 12 conserved amino acid metabolic genes: *Streptococci* spp., 7–10; *C. difficile*, 20; and *B. anthracis*, 39. Simultaneous mutation of a number of tRNA anticodons that bind the specifier loop codons is unlikely to evolve. However, resistance is possible through other means, such as efflux pumps or metabolic inactivation.

## Figures and Tables

**Table 1:** Activity of PKZ18 as an antibiotic. ^1^ Values represent the averages of technical triplicates plus experimental triplicates. MIC: minimum inhibitory concentration values are the lowest compound concentration at which no visible growth was observed after incubation overnight (16 h) [20]. MBC: minimum bactericidal concentration. N/O = not observed, N/D = not determined. ^2^ PKZ18 did not inhibit *E. faecalis* growth completely. At 32 µg/mL, it inhibited growth to half that of the control culture; concentration-dependent growth inhibition exhibited an MBC of 32 µg/mL [20].

Bacteria	MIC ^1^ (MBC) [µg/mL]
**Gram-positive**	
*B. subtilis* (BGSC 1A1)	64 (64)
*B. cereus* (ATCC 7064)	64 (64)
*S. aureus* (ATCC 29213)	64 (N/O)
*E. faecalis* (ATCC 29212)	32 (N/D) ^2^
*S. pyogenes* (clinical isolate)	16 (16)
*S. agalactiae* (clinical isolate)	32 (32)
*S. pneumoniae* (clinical isolate)	16 (16)
*S. mutans* (clinical isolate)	125 (125)
*MRSA* (clinical isolate, pleural fluid)	64 (N/O)
*MRSA* (clinical isolate, blood)	64 (N/O)
**Gram-negative**	
*E. coli* (ATCC 25922)	N/O (N/D)
*P. aeruginosa* (ATCC 27853)	N/O (N/D)
*K. pneumoniae* (clinical isolate)	N/O (N/D)

**Table 2:** PKZ family analogs and activities. Structures of PKZ18 analogs that exclusively disrupt Gram-positive bacterial growth through impeding transcription initiation. None of the analogs inhibited Gram-negative bacterial growth. The different chemical moieties correspond to three locations that were chemically modified: R^1^, R^2^, and R^3^. qRT-PCR confirmed the disruption of aminoacyl-tRNA synthetase gene transcription. Minimum inhibitory concentration (MIC) was evaluated against Gram-positive bacterium *B. subtilis* and methicillin-resistant *Streptococcus aureus* (MRSA) and the control of the Gram-negative *Escherichia coli*.

PKZ18 Family	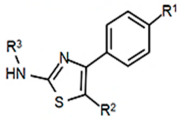	MIC (µg/mL)
PKZ18	** 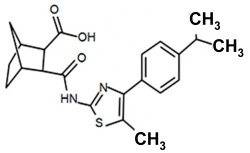 **	64
PKZ18-21	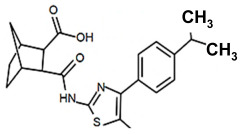	64
PKZ18-22	** 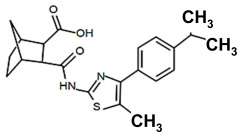 **	32
PKZ18-52	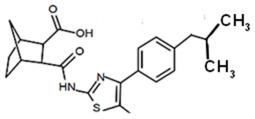	32
PKZ18-53	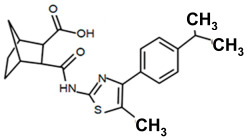	32
PKZ18-54	** 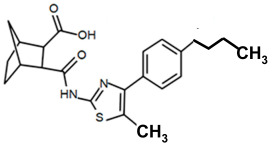 **	128
PKZ18-55	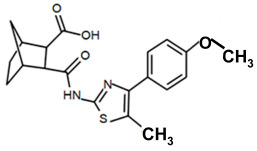	>256
PKZ18-56	** 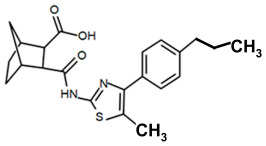 **	>256
PKZ18-57	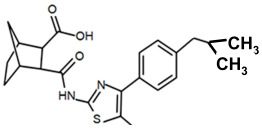	>256

## Data Availability

The data presented in this study are available on request from the corresponding author due to the restrictions of the patent considerations of the institutions. Two U.S. patents have been issued: T-Box Riboswitch-Binding Anti-bacterial Compounds, U.S. Patent 10266527 and Method for Inhibiting Growth of Bacteria, U.S. Patent 11617741.
